# Shear Bond Strength of Zirconia Ceramic to Four Different Core Materials, An *in vitro* Study

**DOI:** 10.30476/DENTJODS.2020.86215.1176

**Published:** 2021-06

**Authors:** Sara Tavakolizadeh, Mohamad Dehghan, Rahab Ghoveizi, DMD, MScD, Anahita Fayyazi

**Affiliations:** 1 Dept. of Prosthodontics, School of Dentistry, Shahid Beheshti University of Medical Sciences, Tehran, Iran; 2 Post Graduate Student of Prosthodontics, Dept. of Prosthodontics, School of Dentistry, Shahid Beheshti University of Medical Sciences, Tehran, Iran

**Keywords:** Composite Resins, CoreRestore, Shear Strength, Yttria Stabilized Tetragonal Zirconia

## Abstract

**Statement of the Problem:**

Different materials can be used to reconstruct the core foundation in all-ceramic restorations. Bond strength of the core material to zirconia is an important factor in long-term restoration success.

**Purpose:**

The aim of this study was to assess shear bond strength (SBS) of zirconia to four different core materials.

**Materials and Method:**

In this experimental *in vitro* study, 40 zirconia ceramic disks (10×3 mm) were prepared and divided to four groups based on core material. Cylinder shaped core specimens (3×4 mm) of non-precious gold alloy (NPG), zirconia ceramic, natural dentin, and composite resin were prepared and bonded perpendicularly to the zirconia disks using Gillmore Needle Apparatus and dual cure resin cement. All samples were thermocycled for 2000 cycles. To evaluate SBS, the specimens were tested by universal testing machine. Data were analyzed using Kruskal-Wallis test followed by Dunn's test with Bonferroni correction. Statistical significance was set at *p*< 0.05.

**Results:**

The highest values for SBS were achieved in composite resin group (11.58±1.74 MPa) followed by NPG (10.32±0.94 MPa), zirconia (7.3±1.11 MPa) and dentin group (6.53±0.56 MPa). SBS in composite resin and NPG core materials were significantly higher than other core materials (*p*< 0.05).

**Conclusion:**

Composite resin and NPG cores showed significant higher bond strength to zirconia in comparison to dentine and zirconia core materials.

## Introduction

There are several methods mentioned in the literature for restoring damaged anterior teeth [ 1]. Increased demand for esthetic restorations and unpredictable biocompatibility of some metal alloys has attracted attention toward metal-free restorations in recent decades [ 2] and has turned them to a routine choice in prosthetic treatments [ 3].

Several materials can be used to compensate the missing tooth structure as a core foundation for extra-coronal restorations. The choice of proper material depends on the amount of remaining tooth structure, esthetics, finances, and treatment duration [ 4]. The non-precious gold alloy (NPG), containing more than 80% copper, first was introduced in 1987. It has optimal physical properties as dental post-cores with simpler preparation and handling compared to nickel-chromium ones [ 5].

In translucent ceramic crown systems, there is concern about the impact of abutment shade beyond the restoration [ 6]. For high translucent restorations with a thickness less than 1.6mm, the shade of underlying abutment may affect the final esthetic result [ 6]. The development of tooth colored post-core systems, such as composite resin or ceramic dowel and core restorations, has improved esthetics [ 7]. Non-metallic post-cores not only provide more esthetic over metallic posts, but also reduce the risk of corrosion and toxicity. Nowadays, many composite resin systems are available specifically designed for core build-up with more fillers, higher strength, and easier manipulation [ 8- 11].

The bond strength of core foundation and crown plays essential role in success rate of full ceramic restorations [ 12]. Previous studies about bond strength of zirconia crowns have been concentrated on surface treatments before cementation and use of various adhesive resins [ 13- 16]. 

The use of airborne particle abrasion technique results in improvement of the bond between resin cement and yttria-stabilized tetragonal zirconia polycrystalline ceramics by producing roughness at the zirconia surface. This surface roughness increases the surface wettability and micromechanical retention with luting agents [ 17].

Several methods including use of phosphoric acid ester monomers like 10-methacryloyloxy-decyl-dihydrogen- phosphate, zirconia coupling agent, and organic silane have been suggested to improve zirconia crowns bond strengths [ 18- 19]. The phosphate containing monomers behave similar to the silane coupling agents because they allow the copolymerization between the methacrylate group and the monomers of a composite resin system. In addition, they bond with the metal oxides in the substrate with phosphoric acid groups. Carboxylic acid is another monomer, which plays an important role in the bond formation [ 17]. High amounts of bond strength were reported in the literature when using methacryloyloxy-decyl-dihydrogen-phosphate- containing resin cement (Panavia F 2.0; Kuraray) [ 20].

Bond strength of zirconia crowns to different core materials has been investigated in limited studies [ 4]. So, the aim of this study was to evaluate shear bond strength (SBS) of zirconia to four different core materials. The null hypothesis of this study was that the core material has no effect on the SBS of zirconia crowns.

## Materials and Method

40 disk samples of multilayer zirconia (Katana Zirconia ML, Kuraray Noritake Dental Inc., Aichi, Japan) were made with 10mm in diameter and 3mm in thickness) using computer-aided design/computer-aided manufacturing technique (CAD/CAM) (Ammangirrbach, Ceramill motion 2, Koblach, Austria). All samples were polished by silicon carbide paper (600 grit matador 991A soflex starcke GmBH&amp;Co., Melle, Germany) and then were mounted in acrylic molds (1×2×4.5 cm) by locating them in the same level of acrylic resin surface. Specimens were sandblasted with 50 µm aluminum oxide particles under 3 bar pressure for 15 seconds from a 10mm distance. All samples were cleaned in ultrasonic bath with 96% propanol for 3 min. Subsequently they were divided into four groups according to the core material tested (N=10).

In the group 1, composite resin cylinders were built up using plastic cylinder (4mm height ×3mm diameter) and were covered with glass slide in order to reach the smooth surface. All the samples were light cured for 40 seconds at power density of 600 MW/cm^2^ with light-emitting diode light-curing unit. In the group 2, NPG alloy cylinders (4mm height ×3mm diameter) were fabricated by cylindrical pattern resin LS molds and casted with NPG alloy by lost wax technique. In the group 3( dentin cylinders), 10 freshly non-carious extracted human third molars were cleaned and disinfected in 0.5% chloramine T solution for 7 days. The roots were cut below the cementoenamel junction using a double-sided diamond disk and cylindrical specimens were obtained using trephine with 3mm inner diameter and 4mm height. In the group 4, zirconia cylinders (4mm height ×3mm diameter) were prepared from Katana zirconia blocks using computer-aided design/computer-aided manufacturing technique. All materials and appliances are described in detail in Table 1.

**Table1:** Materials used in the present study and their composition

Material	Composition	Manufacturer
Katana zirconia	Zirconium oxide	Kuraray Noritake Dental Inc., Japan
	Yttrium oxide	
	Pigments	
NPG	Cu (80.7%), Al (7.8%), Fe (3%), Zn (2.7%), Mn (1.7%), Ni (4.3%)	Aalbadent, USA
Composite resin (3M Filtek™ Z250 Universal Restorative Dental Composite)	BIS-GMA (Bisphenol A diglycidyl ether dimethacrylate), UDMA (urethane dimethacrylate), Bis-EMA (Bisphenol A polyethylene glycol diether dimethacrylate), filled with 60% (volume) silica/zirconia.	Filtek™ Z250, 3M ESPE, St. Paul, MN, USA
Pattern resin LS	Powder: Polymethylmethacrylate, Polyethylmethacrylate, Dibenzoyl peroxide	Self-curing, Acrylic Die Material, GC America
	Liquid: Methylmethacrylate, 2-Hydroxyethyl-Methacrylate	
Panavia F2.0	Paste catalyst: bis-GMA, TEGDMA, glass filler Paste A: silanated silica filler, silanated colloidal silica, MDP, hydrophilic aliphatic D, hydrophobic aliphatic D, dl-camphorquinone, catalysts, initiators	Kuraray, Okayama, Japan
	Paste B: silanated Ba glass, sodium fluoride, hydrophilic aromatic D, hydrophobic aliphatic D, catalysts, accelerators, pigments (filler content. 76.9±0.23 wt%)	
Clearfil ceramic primer plus	Ethanol> 80%, 3-trimethoxysilylpropyl methacrylate< 5%, 10-Methacryloyloxydecyl dihydrogen phosphate	Kuraray, Okayama, Japan
ED Primer II	N-Methacryloyl-5-aminosalicylic acid, water, catalysts, accelerators	Kuraray, Okayama, Japan

All cylindrical specimens were etched with phosphoric acid 37% for 5 seconds, rinsed, and air dried before bonding. Clearfil ceramic primer plus was used to prepare the surface of zirconia disks according to the manufacturer's instructions. After 30 seconds, surface of zirconia disks were gently air dried with oil-free compressed air for 5 sec.

A drop of each bottle A and B of ED Primer II was mixed and applied on the surface of core material specimens by micro brush according to manufacturer’s instruction. After 30 seconds, surface of core specimens were gently air dried with oil-free compressed air for 5sec.

**Figure 1:**
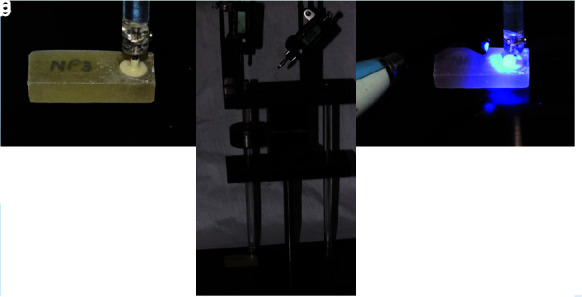
**a:** bonding between zirconia disk and core material with dual cure resin cement; **b:** Use of Gillmore Needle apparatus; **c:** Final curing of resin cement.

Dual resin cement (Panavia F2.0) was used for bonding procedure. Equal amount of each tube A and B of Panavia F2.0 resin cement was mixed for 20 seconds and applied on zirconia surface by spatula. The core material samples were put on the cement surface subsequently (Figure 1a). The cement was allowed to flow under Gillmore Needle Apparatus (Gillmore Needle- 453.6g) (Figure 1b).

Residual amount of cement was removed by a small brush after primary curing for 10 sec. Inhibitor gel (Oxyguard Kurary) was applied for 3 min on the margins of the samples afterward. Finally, all of the samples were cured in four directions for 20 (Figure 1c).

After bonding procedure, all samples were stored in distilled water at 37°C for 24 hours and then thermocycled for 2000 cycles in water bath between 5°C and 55°C with a dwell time of 20 seconds for each temperature with transfer time of 10 seconds.

SBS test was performed using universal testing machine (STM 20 I Santam, Tehran, Iran) with the cross head speed of 0.5 mm/min until bond failure occurred.

The maximal force (MPa) for debonding was recorded. The data was subjected to Kruskal-Wallis test followed by Dunn's test with Bonferroni correction. The significance level was 0.05%.

## Results

Mean ± standard deviation values of SBS for all groups are presented in Table 2.

**Table2:** Descriptive SBS values for different core materials (MPa)

Core material	Minimum	Maximum	Mean (Std. deviation)
Composite resin	8.80	13.89	11.58(1.73)
NPG	8.74	11.70	10.32(0.93)
Zirconia	6.07	9.41	7.29(1.11)
Dentin	6.03	7.49	6.53(0.55)

To investigate the normality of the quantitative variable distribution of data, Kolmogorov Smirnov test was used. With no confirmation of normality assumption, nonparametric analysis of Kruskal-Wallis test and Bonferroni correction were used for comparing the groups. Based on the Kolmogorov-Smirnov test results, the normality of the data distribution in composite resin, NPG, and zirconia except dentin were confirmed. The result of Kruskal-Wallis test showed significant differences between groups (*p*< 0.05) (χ^2^=30.062, *p*< 0.01).

**Figure 2:**
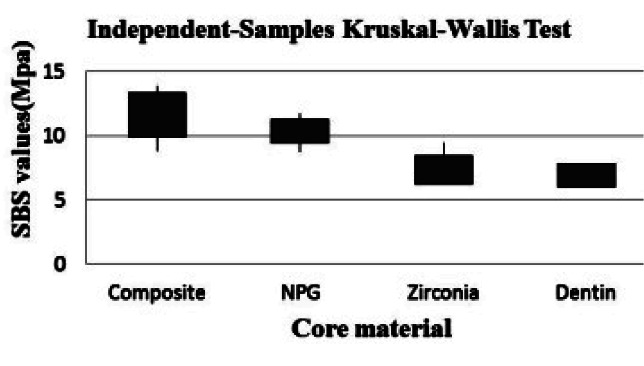
Box plot of SBS (Shear Bond Strength) of Katana zirconia in test groups.

Table 3 shows pairwise comparison of SBS of groups based on Bonferroni correction. There was a significant difference between SBS values of Dentin-NPG, Dentin-Composite, Zirconia-NPG and Zirconia-Composite (*p*< 0.05). Differences between Dentin- Zirconia and NPG-Composite SBS values were not statistically significant (*p*> 0.05). Figure 2 shows that SBS in composite and NPG is higher than zirconia and dentin. 

**Table3:** Pairwise comparison of SBS values for different core materials

Groups	Test Statistic	Std. Error	Std. Test Statistic	Sig.	Adj. Sig.^a^
Dentin-Zirconia	4.800	5.227	0.918	0.358	1.000
Dentin-NPG	19.750	5.227	3.779	0.000	0.001
Dentin-Composite	24.450	5.227	4.678	0.000	0.000
Zirconia-NPG	14.950	5.227	2.860	0.004	0.025
Zirconia-Composite	19.650	5.227	3.759	0.000	0.001
NPG-Composite	4.700	5.227	0.899	0.369	1.000

Each row tests the null hypothesis that the group’s distributions are the same. Asymptotic significances (2-sided tests) are displayed. The significance level is 0.05.

a. Significance values have been adjusted by the Bonferroni correction for multiple tests.

## Discussion

Selection of an appropriate material in restoring the core of a damaged tooth depends on the remaining dental structure and esthetic factors [ 4]. Proper bonding between the core material and the crown guarantees the long term success of a restoration [ 4]. Recently, zirconia crowns have gained lots of popularity among dentists due to the high strength and esthetic [ 21- 22].

Bonding of zirconia to the core materials is challenging. Chemical structure contains polycrystalline and lack of amorphous glass phase is believed to be the main cause of weak bond of yttria-stabilized tetragonal zirconia polycrystalline ceramics [ 17]. Several methods have been suggested to improve bonding properties of zirconia, including conventional surface treatments like grinding with diamond burs, air abrasion with aluminum oxide (Al2O3), tribochemical silica-coating, acid etching with hydrofluoric acid, coupling with silane agent, plasma spraying with hexamethyldisiloxane, and combinations of any of these methods [ 23- 32]. Jiao *et al*. [ 33] mentioned that sandblasting of zirconia increased SBS between zirconia and resin cement. So, in this study all specimens were sandblasted with 50µ aluminum oxide under pressure of 3 bars for 15 seconds as noted in similar study [ 4]. Our results showed significantly higher SBS of zirconia to composite core material and NPG, witch rejecting our null hypothesis. In order to justify these results, we can refer to Al-Harbi *et al*. [ 17], which evaluated SBS of yttria-stabilized tetragonal zirconia polycrystalline ceramics to different core materials with the use of three primer/resin cement systems. They pointed out that formation of more surface oxides in metal and composite resin cores might be a cause for stronger SBS in these materials compared with those formed on the surface of the zirconia cores [ 17].

The high organic content and moisture of natural dentin could be the main reasons of weak bond between zirconia and dentin core, these findings are in agreement with other studies [ 34- 35]. Although dentin bond strength is lower than other core materials, it does not mean the bond is insufficient [ 4]. In cases which the dental structure is sufficient for placement of a zirconia crown, the tooth structure should not be removed to create stronger bond to the zirconia crown.

Frattes *et al*. [ 4] reported that metallic alloys and zirconia core materials had significantly higher SBS compared to dentin. The SBS value of zirconia core in Frattes *et al*. [ 4] study was higher than ours. This difference may be attributed to the different methods of air abrasion before cementation. Variables like particle size and shape, incidence angle, moisture and pressure of air abrasion system might play an important role in SBS values [ 27]. Although zirconia showed higher SBS values than dentin in our research, this difference was not proved to be statistically important.

Similar to our findings, Al-Harbi *et al*. [ 17] showed the mean zirconia SBS to metal (nickel-chromium) and composite core material were higher than zirconia core and there was no significant difference between nickel-chromium and composite. This study showed higher SBS of composite resin cores compare to zirconia cores, and there was no significant difference between composite and NPG SBS. Copolymerization between the cement monomers and the composite resin at the interface probably results in greater bond strength of the composite resin core material [ 4]. Due to the same SBS of composite resin and metal cores when bonded to zirconia crown, using composite core in esthetic zone seems reasonable. 

Prior to bonding, Clearfil ceramic primer was used to prepare zirconia disk surfaces according to the manufacturer's instructions. This primer contains 3-methacryloxy propyl trimethoxy silane mixed with 10-methacryloyloxy-decyl-dihydrogen-phosphate monomer. By using this primer, chemical bond is formed between zirconia and aluminum oxide substrate and the phosphate ester group of this monomer [ 36- 38]. These findings are in agreement with other studies [ 17].

This study was an *in vitro* study. Confounding factors like masticatory forces, salivary flow, and fatigue should also be considered to stimulate oral cavity condition and obtain more valid and reliable results for clinical practice. There are few studies about the effect of different types of core material on the SBS values of zirconia crowns. Therefore, more studies with different core materials, bonding systems, aging process and debonding forces is recommended.

## Conclusion

Regarding the limitation of this study, the effect of different core materials on the SBS of zirconia was significant. Considering the importance of bonding between crown and core, composite and NPG could be recommended as foundation core material.
